# Anti-hypertensive medications and injurious falls in an older population of low socioeconomic status: a nested case-control study

**DOI:** 10.1186/s12877-018-0871-7

**Published:** 2018-08-28

**Authors:** Zafirah Banu, Ka Keat Lim, Yu Heng Kwan, Kai Zhen Yap, Hui Ting Ang, Chuen Seng Tan, Warren Fong, Julian Thumboo, Kheng Hock Lee, Truls Ostbye, Lian Leng Low

**Affiliations:** 1 Department of Pharmacy, Faculty of Science, National University of Singapore, Singapore, Republic of Singapore; 2 Program in Health Services and Systems Research, Duke-NUS Medical School, Singapore, Republic of Singapore; 3 Saw Swee Hock School of Public Health, National University of Singapore and National University Health System, Singapore, Republic of Singapore; 4 Department of Family Medicine and Continuing Care, Singapore General Hospital, Singapore, Republic of Singapore; 5 Department of Rheumatology and Immunology, Singapore General Hospital, Singapore, Republic of Singapore; 6 Duke-NUS Medical School, Singapore, Republic of Singapore; 7 Department of Medicine, Yong Loo Lin School of Medicine, National University of Singapore, Singapore, Republic of Singapore

**Keywords:** Aged, Antihypertensive agents, Falls, Socio-economic status

## Abstract

**Background:**

This study aimed to determine whether the number of anti-hypertensive medication classes or any change in anti-hypertensive medication were associated with injurious fall among the community-dwelling older population of low socioeconomic status.

**Methods:**

Using data from electronic medical records, we performed a nested case-control study among older Singapore residents (≥60) of low socioeconomic status (*N* = 210). Controls (*n* = 162) were matched to each case (*n* = 48) by age and gender. Variables with *p* < 0.10 in univariate analysis were included in multivariate analysis. We used conditional logistic regression to assess the associations of the number of anti-hypertensive medication classes and change in anti-hypertensive medication with injurious falls. We also performed stepwise regressions as sensitivity analyses. *p* < 0.05 was considered statistically significant.

**Results:**

The mean (±SD) age of participants was 78.1 (± 8.33) years; 127 (60.4%) were female, 189 (90.0%) were Chinese. Those on ≥2 anti-hypertensive medication classes had an increased risk of experiencing an injurious fall compared to those not on any anti-hypertensive medication (OR = 5.45; CI:1.49–19.93; *p* = 0.01). Among those who were taking anti-hypertensive medication, those who had a change in the medication 180-day prior to injurious fall had a significantly increased risk of experiencing an injurious fall compared to those that did not report any change in anti-hypertensive medication (OR = 3.88; CI:1.23–12.19; *p* = 0.02). Sensitivity analyses generated consistent findings.

**Conclusion:**

Both ≥2 anti-hypertensive medication classes and change in anti-hypertensive medication were associated with an increased risk of experiencing an injurious fall among the older population of low socioeconomic status. Our findings could guide prescribers to exercise caution in the initiation of anti-hypertensive medications or in making medication changes, especially among the older population of low socioeconomic status.

**Electronic supplementary material:**

The online version of this article (10.1186/s12877-018-0871-7) contains supplementary material, which is available to authorized users.

## Background

Falling is a common and severe problem faced by the older population worldwide with 28–35% of community-dwelling older adults > 64 years and 32–42% of those > 70 years estimated to experience at least one fall each year [1]. This has significant social, economic and health implications. Falls result in increased morbidity and mortality, with approximately 10% of falls leading to serious injuries, such as fractures, intracranial bleeding, serious lacerations and even death [2]. Besides higher healthcare utilization due to falls-related injuries [3], older adults may also experience psychological damages that result in self-imposed limitation in mobility, increased dependency on others for activities of daily living, depression and anxiety [4]. This leads to higher psychological and economic burden for the caregivers and the country [5].

While numerous studies have examined the association between anti-hypertensive medications and falls, most studies only focused on specific classes of anti-hypertensive. Existing systematic reviews and meta- analyses of these studies found weak or no association between the different anti-hypertensive classes and falls [6–8].

Recent evidence suggests that intensity of treatment [9, 10] or changes in anti-hypertensive medication [11] may be associated with falls. This is plausible as anti-hypertensive treatment intensity and changes in anti-hypertensive medication have been associated with orthostatic hypotension [12] and neurological side effects [11] which could lead to falls.

Our study aimed to examine whether a higher number of anti-hypertensive medication classes or a recent change in anti-hypertensive medication was associated with injurious falls, defined as falls that required medical services. The focus on injurious falls is due to its direct implication on healthcare utilization, compared to any fall occurrence commonly examined in previous studies [13]. We were interested in studying the older adults of low socioeconomic status (SES) because they are at higher risk of falls [14] and suffer from a higher burden of hypertension [15, 16]. As such, our findings could guide prescribers to exercise extra precaution in the initiation of anti-hypertensive medications or in making medication changes, especially among the older adults of low SES.

## Methods

### Study design

We conducted a nested case-control study involving participants (*N* = 264) taking part in the Integrated Community of Care (ICoC) programme [17], a novel care model in Singapore General Hospital (SGH) that integrates hospital-based transitional care with health and social care in the community. This ongoing programme targets older adults (≥ 60 years old) living in public rental flats, which offer heavily subsidized rents for the underprivileged population, among whom 88% earn less than S$670/month (USD498.12 based on SGD1: USD0.74 conversion) [18]. Participants were included in the ICoC programme if they had at least one inpatient and/or outpatient encounter with SGH 3 years prior to entry into the ICoC programme. For those with dementia, they were included if they were capable of independent living or had a caregiver. This study was reviewed and approved by the SingHealth Centralized Institutional Review Board. Written informed consent was obtained for every participant.

We defined cases as participants who experienced any injurious falls from August 2014 to August 2017. For those with multiple injurious falls, we collected information on their baseline characteristics from the most recent injurious fall. For each case, up to 8 controls who had no recorded injurious falls within the same time period were matched on age (±5 years) and gender. For each control, the fall date of their matched case was taken as the baseline to obtain the relevant independent variables (e.g., medications) 180-day prior to the fall date.

To examine the association between the number of anti-hypertensive medication classes and injurious falls, we identified 48 participants who experienced an injurious fall and 162 matched controls who did not experience any injurious falls during the 3-year period. An average of 3 controls (minimum = 1; maximum = 8) were matched to each case on age (±5 years) and gender.

To examine the association between a change in anti-hypertensive medication and injurious fall, we used the same matching but restricted our sample to include only those who took anti-hypertensive medication. In total, 38 cases and 101 controls were included in this analysis (*N* = 139). An average of 2 controls (minimum = 1; maximum = 8) were matched to each case on age ± 5 years) and gender.

### Dependent variable of interest

We defined an injurious fall as a fall that required medical services either in the emergency department or inpatient hospitalisation in SGH. This could be minor injuries such as those that required only dressing and wound cleaning or major injuries such as those that required surgery, casting or resulted in neurological or internal injury.

### Exposure variables of interest

We categorized the number of anti-hypertensive medication classes consumed during the 180-day prior to the fall date into 3 categories: None, 1 class and ≥ 2 classes of anti-hypertensive medications.

We considered any anti-hypertensive medications from the following classes – angiotensin converting enzyme inhibitors (ACEI), angiotensin receptor blockers (ARB), beta blockers (BB), calcium channel blockers (CCB) and diuretics. Specifically, the older adults in our sample took enalapril, captopril or lisinopril as ACEI; losartan, valsartan, telmisartan or irbesartan as ARB; atenolol or bisoprolol as BB. amlodipine or slow-release nifedipine as CCB; hydrochlorothiazide, frusemide or spironolactone as diuretics.

We defined a change in anti-hypertensive medication as an addition of a new class of anti-hypertensive medication, an increase in the dosage of the existing medication or a switch to a new class of anti-hypertensive medication. No participants in our study experienced a reduction in dosage or in number of anti-hypertensive medications. Participants who reported no change in anti-hypertensive medication were those who had the same anti-hypertensive medications throughout the 180-day period prior to the fall date or matched fall date.

### Data collection

We reviewed existing literature to determine the information to be collected. All data were collected from SGH medical records for patients, in accordance to the consent provided by the ICoC participants.

For each participant, we collected information on age, gender, body mass index, race, blood pressure and Morse Fall Scale score at the time of the fall. All other baseline characteristics of participants were based on the 180-day prior to the fall date or matched fall date. Information on visual or hearing impairment, prior smoking and alcohol history was also obtained. Cognitive impairment was measured using the Abbreviated Mental Status Test [19] or the Short Portable Mental Status Questionnaire [20]. Both tests consist of 10 items appraising orientation, memory, calculation and facial recognition. The cut-offs for these 2 tests were adjusted according to age and education. Our measure of co-morbidity was the Charlson’s Comorbidity Index (CCI) [21], an indicator based on diagnoses of 17 diseases, including myocardial infarction, diabetes and cerebrovascular disease.

Fall risk was assessed using the Morse Fall Scale based on a patient’s history of falling, presence of > 1 medical diagnosis, use of ambulatory aids such as a cane, wheelchair, or walking frame, insertion of intravenous/heparin lock, types of gait and mental status. The scale was validated in Singapore [22] with participants scoring ≥55 considered to be at a high risk of falling. As such, we categorised participants into 2 categories: with and without a high fall risk.

Lastly, we recorded prescription drug name, class, dose, frequency and any change in anti-hypertensive medications during the 180-day period prior to the most recent fall date. Besides antihypertensive medications, falls could also be attributed to other medications such as psychotropic medications and statins. Psychotropic medications could lead to falls by causing sedation, orthostatic hypotension, confusion, slow reaction times and impaired balance [23] whereas statin therapy could result in a decline in muscle strength [24]. Thus, we also recorded the use of statin and psychotropic medication 180-day prior to the fall date. Psychotropic medications included antidepressant, antipsychotic, sedative-hypnotic, antiepileptic, anti-Parkinson’s medication, or narcotic.

We defined polypharmacy as the use of ≥4 chronic medications [25]. We recorded details of all medications dispensed for a period of ≥3 months. This included prescription, non-prescription and over-the-counter (OTC) medication but excluded anti-hypertensive medication to avoid double-counting. We considered fixed-dose combinations (multiple active ingredients in one preparation) as a single medication because we were interested in looking at the pill count rather than the total classes of medication. A higher pill count results in poorer medication adherence which has been associated with an increased rate of falls among older adults [26].

### Statistical analysis

We first summarized the baseline characteristics of participants using means and standard deviations (SDs) or frequencies and percentages. We compared participants who did with those who did not experience any injurious fall using t-tests or Wilcoxon rank-sum tests for continuous variables and Chi-squared or Fisher’s exact tests for categorical variables.

Subsequently, we performed conditional logistic regression, using injurious fall as the dependent variable. We began with univariate regression and independent variables with *p* < 0.10 were included in the multivariate regression models. We performed two multivariate conditional logistic regressions, using the number of anti-hypertensive medication classes or any change in anti-hypertensive medication as the exposure variables respectively. Associations with *p* < 0.05 in the multivariate regressions were regarded as statistically significant. To examine whether the results were consistent in parsimonious models, we also performed sensitivity analyses by including variables with *p* < 0.10 into both forward and backward stepwise regressions. We specified *p* > 0.10 for removal from the model and *p* < 0.05 for addition to the model. We examined all final models for collinearity using variance inflation factor (VIF). All analyses were performed using STATA SE version 14.0 (StataCorp College Station, Texas).

## Results

### Participant characteristics

Table 1 presents the demographic and clinical characteristics of both cases and controls. The mean (±SD) age of the 210 participants was 78.1 (± 8.33) years; 127 (60.4%) were female, 189 (90.0%) were Chinese. Of the 48 participants who experienced an injurious fall, 44 (91.2%) experienced minor injuries and 4 (8.33%) experienced major injuries.

**Table 1:** Baseline characteristics of study participants

Characteristics	Total sample (*n* = 210)	Cases (*n* = 48)	Controls (*n* = 162)	*P*-value
Age, years, mean ± SD	78.1 ± 8.3	79.5 ± 8.4	77.7 ± 8.3	0.18
Female, *n* (%)	127 (60.4)	32 (66.7)	95 (58.6)	0.32
Body mass index, kg/m^2^, mean ± SD	23.9 ± 5.2	24.8 ± 6.0	23.7 ± 4.8	0.40
Chinese^c^, *n* (%)	189 (90.0)	46 (95.3)	143 (88.3)	0.17
SBP on admission, mmHg, mean ± SD	133.1 ± 19.2	137.7 ± 23.7	131.7 ± 17.7	0.07
DBP on admission, mmHg, mean ± SD	68.3 ± 10.3	69.3 ± 11.8	67.9 ± 9.9	0.78
Visual impairment, *n* (%)	29 (13.8)	10 (20.8)	19 (11.7)	0.15
Hearing impairment, *n* (%)	18 (8.6)	5 (10.4)	13 (8.02)	0.57
Use of walking aid, *n* (%)	84 (40.0)	37 (77.1)	47 (29.0)	< 0.01
High risk of fall^a^, *n* (%)	42 (20.0)	21 (43.8)	21 (13.0)	< 0.01
Smoking history, *n* (%)	48 (22.9)	10 (20.8)	38 (23.4)	0.70
Alcohol history, *n* (%)	23 (11.0)	5 (10.4)	18 (11.1)	0.90
Medical history, *n* (%)				
Hypertension	162 (77.1)	37 (77.1)	125 (77.2)	0.88
Cerebrovascular accident or transient ischaemic attack	39 (18.6)	12 (25.0)	27 (16.7)	0.19
Ischaemic heart disease	52 (24.8)	12 (25.0)	40 (24.7)	0.97
Diabetes mellitus	75 (35.7)	18 (37.5)	57 (35.2)	0.79
Hyperlipidaemia	147 (70.0)	33 (68.8)	114 (70.4)	0.83
Osteoporosis	20 (9.5)	5 (10.4)	15 (9.3)	0.78
Cancer	26 (12.4)	7 (14.5)	19 (11.7)	0.60
Cognitive impairment	21 (10.0)	6 (12.5)	15 (9.3)	0.59
Prior history of falls, *n* (%)	49 (23.3)	18 (37.5)	31 (19.1)	0.01
Charlson Comorbidity Index, mean ± SD	5.1 ± 2.3	6.3 ± 2.5	4.7 ± 2.1	< 0.01
Medication use, *n* (%)				
Statin	124 (59.0)	29 (60.4)	95 (58.6)	0.83
Psychotropic medication	36 (17.1)	13 (27.1)	23 (14.2)	0.04
Anti-hypertensive medication	148 (70.4)	40 (83.3)	108 (66.7)	0.03
≥ 2 anti-hypertensive medication	72 (34.3)	31 (64.6)	41 (25.3)	< 0.01
Any change in anti-hypertensive medication^b^	41 (19.5)	20 (41.7)	21 (13.0)	< 0.01
Type of anti-hypertensive, *n* (%)				
Angiotensin-converting enzyme inhibitor	43 (20.5)	15 (31.3)	28 (17.3)	0.04
Angiotensin II receptor blocker	41 (19.5)	15 (31.3)	26 (16.0)	0.02
Beta-blocker	56 (26.7)	21 (43.8)	35 (21.6)	< 0.01
Calcium channel blocker	75 (35.7)	17 (35.4)	58 (35.8)	0.94
Diuretic	24 (11.4)	12 (25.0)	12 (7.41)	< 0.01
Polypharmacy, *n* (%)	109 (51.9)	32 (66.7)	77 (47.5)	0.02

*SD* Standard deviation, *SBP* Systolic blood pressure, *DBP* Diastolic blood pressure

^a^High risk of fall = Defined using Morse Fall Scale risk score of 55 or more

^b^Any change in anti-hypertensive medication = An addition of a new class of anti-hypertensive medication or an increase in the dosage of the existing medication or a switch to a new class of anti-hypertensive medication; Polypharmacy = Use of 4 or more chronic medication

Reference group:

^c^Non-Chinese

Majority of the participants (*n* = 148, 70.4%) were on anti-hypertensive medication. Among anti-hypertensive medication users, 76 (51.3%) took 1 class of medication and 72 (48.6%) took ≥2 classes of medications. No participants in our study took fixed-dose combinations of anti-hypertensive medication. In addition, 41 (27.7%) participants had a change in anti-hypertensive medication within the 180-day period prior to the fall.

Participants who experienced an injurious fall reported a higher risk of fall, greater use of psychotropic medication, a higher rate of previous falls and a higher proportion with polypharmacy. They were more likely to use walking aids and have a higher mean CCI compared to those that did not experience any injurious fall. In addition, they reported greater use of ACEI, ARB, BB and diuretics.

### Association between the number of anti-hypertensive medication classes and injurious falls

In univariate analysis (Table 2), five variables were associated *(p < 0.10)* with the occurrence of injurious falls: Charlson’s Comorbidity Index (OR:1.33; 95% CI:1.14–1.56; *p* < 0.001), high fall risk (OR:5.69; 95% CI:2.42–13.36; *p* < 0.001), visual impairment (OR:2.48; 95% CI:1.01–6.08; *p* = 0.05), exposure to psychotropic medication (OR:2.21; 95% CI:0.97–5.06; *p* = 0.06) and polypharmacy (OR:2.22; 95% CI:1.08–4.54; *p* = 0.03). These variables were included as predictors in the multivariate model (Table 2), which showed a significant association between the number of anti-hypertensive medication classes and the odds of experiencing an injurious fall (OR = 5.45; 95% CI:1.49–19.93; *p* = 0.01). VIF for each independent variable in the final model was < 5. While walking aid and prior history of falls had *p* < 0.10 in the univariate analyses, they were not included in the multivariate model as they were part of the high fall risk, which we had already adjusted for.

**Table 2:** Association between number of anti-hypertensive medication and injurious falls (N = 210)

	Unadjusted OR (95% CI)	P-value	Adjusted OR (95% CI)	P-value
Number of anti-hypertensive medication				
0	1.00		1.00	
1	0.92 (0.30–2.87)	0.89	2.10 (0.48–9.18)	0.33
≥ 2	4.75 (1.77–12.72)	< 0.01	5.45 (1.49–19.93)	0.01
Charlson comorbidity index	1.33 (1.14–1.56)	< 0.01	1.26 (1.04–1.54)	0.02
High risk of fall^a^	5.69 (2.42–13.36)	< 0.01	7.21 (2.37–21.94)	< 0.01
Visual impairment	2.48 (1.01–6.08)	0.05	2.02 (0.70–5.86)	0.19
Exposure to psychotropic medication	2.21 (0.97–5.06)	0.06	1.12 (0.38–3.37)	0.83
Polypharmacy	2.22 (1.08–4.54)	0.03	1.29 (0.49–3.43)	0.61
Age (years)	0.92 (0.82–1.04)	0.19		
BMI (kg/m^2^)	1.06 (0.98–1.13)	0.13		
Chinese^c^	2.99 (0.62–14.34)	0.17		
SBP on admission (mmHg)	1.01 (0.99–1.03)	0.13		
DBP on admission (mmHg)	1.01 (0.98–1.04)	0.52		
Hearing impairment	1.27 (0.41–3.92)	0.67		
Use of walking aid^b^	8.18 (3.29–20.31)	< 0.01		
Smoking history	1.06 (0.42–2.67)	0.90		
Alcohol history	1.13 (0.34–3.74)	0.84		
Medical history				
Hypertension	1.05 (0.47–2.38)	0.90		
Cerebrovascular accident or transient ischaemic attack	1.97 (0.87–4.49)	0.11		
Ischaemic heart disease	1.26 (0.57–2.76)	0.56		
Diabetes mellitus	1.17 (0.60–2.28)	0.65		
Hyperlipidaemia	0.91 (0.44–1.89)	0.81		
Osteoporosis	1.05 (0.33–3.32)	0.93		
Cancer	1.28 (0.48–3.46)	0.62		
Cognitive impairment	1.33 (0.47–3.80)	0.59		
Prior history of falls^b^	2.51 (1.18–5.37)	0.02		
Statin use	0.88 (0.45–1.71)	0.71		

*OR* Odds ratio, *CI* 95% confidence interval, *SBP* Systolic blood pressure, *DBP* Diastolic blood pressure

^a^High risk of fall = Defined using Morse Fall Scale risk score of 55 or more; Polypharmacy = Use of 4 or more chronic medication

^b^Even though these variables had p < 0.10 in univariate analysis, they were not included in the multivariate analysis as these variables were part of the high fall risk, which we had already adjusted for

Reference group:

^c^Non-Chinese

Apart from the exposure variable, the only other variables that reached statistical significance in the multivariate model were Charlson’s Comorbidity Index (OR:1.26; 95% CI:1.04–1.54; *p* = 0.02) and high fall risk (OR:7.21; 95% CI:2.37–21.94; *p* = 0.001). However, the odds ratio of those taking only 1 class of anti-hypertensive medication (OR:2.10; 95 CI:0.48–9.18; *p* = 0.33) was not significantly associated with experiencing an injurious fall.

In sensitivity analyses, both forward and backward stepwise regressions yielded identical models (Additional file 1: Table S1). Those on ≥2 anti-hypertensive medication classes had increased odds of experiencing an injurious fall compared to those with no anti-hypertensive medication. The odds ratio was consistent with the main analyses, indicating that our findings were robust. VIF for each independent variable in stepwise regression model was < 5.

### Association between a change in anti-hypertensive medication and injurious falls

In our univariate analysis (Table 3), 3 variables were associated *(p < 0.10)* with the occurrence of injurious falls: Charlson’s Comorbidity Index (OR:1.28; 95% CI:1.08–1.51; *p* = 0.004), high fall risk (OR:6.32; 95% CI:2.16–18.46; *p* = 0.001) and a history of hypertension (OR:0.28; 95 95% CI:0.07–1.21; *p* = 0.09). After adjusting for Charlson’s Comorbidity Index, high fall risk and history of hypertension, any change in anti-hypertensive medication within the 180-day period prior was associated with higher odds of experiencing an injurious fall (OR = 3.88; 95%CI:1.23–12.19; *p* = 0.02). VIF for each independent variable in the final model was < 5.

**Table 3:** Association between change in anti-hypertensive medication and injurious fall (N = 139)

	Unadjusted OR (95% CI)	P-value	Adjusted OR (95% CI)	P-value
Any change in anti-hypertensive medication^a^				
No	1.00		1.00	
Yes	5.66 (2.12–15.15)	< 0.01	3.88 (1.23–12.19)	0.02
Charlson comorbidity index	1.28 (1.08–1.51)	< 0.01	1.28 (1.04–1.58)	0.02
High risk of fall^b^	6.32 (2.16–18.46)	< 0.01	5.73 (1.60–20.51)	0.01
Visual impairment	2.10 (0.74–6.01)	0.17		
Exposure to psychotropic medication	2.07 (0.78–5.49)	0.14		
Polypharmacy	1.83 (0.80–4.19)	0.15		
Age (years)	0.90 (0.78–1.04)	0.16		
BMI (kg/m^2^)	1.05 (0.97–1.14)	0.21		
Chinese^d^	2.10 (0.43–10.42)	0.36		
SBP on admission (mmHg)	1.00 (0.99–1.03)	0.99		
DBP on admission (mmHg)	1.00 (0.96–1.04)	0.36		
Hearing impairment	0.55 (0.65–4.75)	0.59		
Use of walking aid^c^	18.07 (4.15–78.65)	< 0.01		
Smoking history	1.31 (0.45–3.76)	0.62		
Alcohol history	0.85 (0.17–4.31)	0.85		
Medical history				
Hypertension	0.28 (0.07–1.21)	0.09	0.30 (0.05–1.75)	0.18
Cerebrovascular accident or transient ischaemic attack	1.50 (0.63–3.59)	0.36		
Ischaemic heart disease	0.89 (0.39–2.07)	0.79		
Diabetes mellitus	0.99 (0.46–2.17)	0.99		
Hyperlipidaemia	0.77 (0.27–2.19)	0.62		
Osteoporosis	1.52 (0.44–5.20)	0.50		
Cancer	1.76 (0.61–5.09)	0.30		
Cognitive impairment	1.00 (0.29–3.45)	1.00		
Prior history of falls	1.84 (0.79–4.30)	0.16		
Statin use	0.50 (0.21–1.18)	0.12		

*OR* Odds ratio, *CI* 95% confidence interval, *SBP* Systolic blood pressure, *DBP* Diastolic blood pressure

^a^Any change in anti-hypertensive medication = An addition of a new class of anti-hypertensive medication or an increase in the dosage of the existing medication or a switch to a new class of anti-hypertensive medication

^b^High risk of fall = Defined using Morse Fall Scale risk score of 55 or more.; Polypharmacy = Use of 4 or more chronic medication

^c^Even though this variable had p < 0.10 in univariate analysis, it was not included in the multivariate analysis as these variables were part of the high fall risk, which we had already adjusted for

Reference group:

^d^Non-Chinese

In our sensitivity analyses, both the forward and backward stepwise regressions yielded identical models (Additional file 1: Table S2). Those who had a recent change in anti-hypertensive medication had higher odds of experiencing an injurious fall compared to those with no change in anti-hypertensive medication (OR:3.66; 95 CI:1.19–11.23; *p* = 0.01). The odds ratio was consistent with that our main analyses, implying that our findings were robust. VIF for each independent variable in the stepwise regression model was < 5.

## Discussion

We found that older adults with low SES on ≥2 anti-hypertensive medication classes had higher odds of experiencing an injurious fall compared to those without any anti-hypertensive medication. Among older adults with anti-hypertensive medication, those with a recent change in anti-hypertensive medication also had increased odds of experiencing an injurious fall. These findings contribute to the literature as most existing studies examined the association between anti-hypertensive classes and falls among general community dwelling older adults [13].

Our findings were consistent with an earlier study [9] which quantified treatment intensity using defined daily dose (DDD) and examined any falls as the outcome. Meanwhile, Tinetti et al. that also examined the number of anti-hypertensive medication classes [10] like our study found no association between it and injurious falls. This difference in results may be attributable to different study samples as our study examined older adults of low SES who are at higher risk of falls whereas Tinetti et al. examined general community-dwelling older adults [10]. To illustrate it in local context, the proportion of those with hypertension (162/210 = 77.1%) and those who experienced falls (48/210 = 22.9%) were higher in our study cohort compared to the general older population (73.9%, 8.2% respectively) in Singapore [27, 28]. Concurrent administration of ≥2 anti-hypertensive medication classes may increase risk of experiencing an injurious fall by exacerbating postural hypotension or symptoms of dizziness and fatigue [12].

Our literature search only retrieved one study examining the association between a change in anti-hypertensive medication and the risk of experiencing an injurious fall [11]. The study found that long term addition or titration of anti-hypertensive medication was not associated with serious fall injuries. We also included those who switched to a different class of anti-hypertensive medication in our analysis and this might have contributed to the increased risk of experiencing an injurious fall in our study. A change in medication may induce electrolyte disturbances which consequently impairs balance and gait [11] and makes older adults more susceptible to falls.

Our findings have several clinical implications. It is important to be wary of the number of anti-hypertensive medications prescribed to the older population as some older people may be taking more anti-hypertensive medication than needed [29]. Prescribers should develop patient-specific treatment goals and avoid “over-treatment” of hypertension, particularly among older population of low SES. A recent study by Zia et al. [30] showed that the consumption of two or more fall risk increasing drugs (FRID) is a significant predictor for falls. Anti-hypertensive medication was identified to be one of the many FRIDs. As such, it is imperative that healthcare providers discuss risk and preventative strategies for falls with patients when commencing anti-hypertensive medications or when adding, switching or increasing the dose of anti-hypertensive medications.

The findings from this study should be interpreted within the context of the following limitations. We defined “injurious falls” based on healthcare use [31], specifically falls requiring medical services as we were concerned about healthcare utilization resulting from falls. We acknowledge that there are other definitions of “injurious falls” in literature [31] such as those based on symptoms only (e.g. fractures) or a combination of symptoms and healthcare use. Therefore, our findings may not be generalizable to other definitions of “injurious falls”. We checked for confounding for a wide array of conditions and medications associated with falls and adjusted for the appropriate variables in multivariate analyses. However, we acknowledge there is still a possibility of residual confounding as our study lacked information on important variables such as frailty and blood pressure. Previous study [32] found substantial differences in the associations between cardiovascular medication use and fall risk in frail and robust older adults. In addition, as blood pressure measurements immediately prior to falls were not available, we were unable to ascertain whether the falls were due to orthostatic hypotension resulting from anti-hypertensive medications.

We also did not collect any measures of adherence to anti-hypertensive medication. Nevertheless, we note that non-adherence would result in underestimation of a true effect. We did not consider the dose of each anti-hypertensive medication. As such, we could not attribute our findings to the dose intensity of each medication. Due to the small samples, our analyses were unable to account for specific type and class of anti-hypertensive medications. Lastly, although the small sample size and the small number of injurious fallers in our study may have posed some challenges, such as imprecise estimates and potentially the sample is not representative of the population, our findings from both the main and sensitivity analyses were consistent.

## Conclusion

In a nutshell, our study demonstrated that ≥2 anti-hypertensive medication classes and a change in anti-hypertensive medication were associated with an increased risk of injurious falls among older adults of low SES. Prescribers should design patient-specific treatment goals to prevent the “over-treatment” of hypertension. In addition, the risk of and preventative strategies for falls should be discussed with patients when commencing anti-hypertensive medications or when adding, switching or increasing the dose of anti-hypertensive medications, especially among older adults of low SES who are more prone to falling.

## Additional file

Additional file 1: Results of sensitivity analyses. The file contains two tables (**Tables S1 and S2**) presenting the results of the sensitivity analyses (stepwise regression). (DOCX 17 kb)

## Abbreviations

ACEi: Angiotensin converting enzyme inhibitors
ARB: Angiotensin receptor blockers
BB: Beta blockers
CCB: Calcium channel blockers
CCI: Charlson’s co-morbidity index
DDD: Defined-daily dose
FRID: Fall risk increasing drugs
ICoC: Integrated Community of Care programme
OR: Odds ratio
OTC: Over-the-counter
SD: Standard deviation
SES: Socio-economic status
SGD: Singapore Dollar
SGH: Singapore General Hospital
USD: United States Dollar
VIF: Variance inflation factor
