# Supplementary material for: Anti-hypertensive medications and injurious falls in an older population of low socioeconomic status: a nested case-control study
Source: BMC Geriatr. 2018 Aug 28;18:195. doi: 10.1186/s12877-018-0871-7 (PMC6114512; doi:10.1186/s12877-018-0871-7)
Supplement: Supplementary file 1 — Results of sensitivity analyses. The file contains two tables (Tables S1 and S2) presenting the results of the sensitivity analyses (stepwise regression). (DOCX 17 kb) [file 12877_2018_871_MOESM1_ESM.docx]

**ADDITIONAL FILE**

**Table S1 Sensitivity Analysis (Stepwise regression)**

| **Association between the number of anti-hypertensive medication classes and injurious falls (N = 210)** | | | | |
| --- | --- | --- | --- | --- |
|  | Adjusted OR  (95% CI) | P-value | Stepwise Regression^a^  (95% CI) | P-value |
| Number of anti-hypertensive medication |  |  |  |  |
| 0 | 1.00 |  | 1.00 |  |
| 1 | 2.10 (0.48-9.18) | 0.33 | 1.87 (0.46-7.56) | 0.37 |
| ≥ 2 | 5.45 (1.49-19.93) | 0.01 | 5.37 (1.60-18.12) | 0.01 |
| Charlson comorbidity index | 1.26 (1.04-1.54) | 0.02 | 1.29 (1.07-1.54) | 0.01 |
| High risk of fall^b^ | 7.21 (2.37-21.94) | <0.01 | 7.06 (2.44-20.45) | <0.01 |
| Visual impairment | 2.02 (0.70-5.86) | 0.19 |  |  |
| Exposure to psychotropic medication | 1.12 (0.38-3.37) | 0.83 |  |  |
| Polypharmacy | 1.29 (0.49-3.43) | 0.61 |  |  |
|  |  |  |  |  |
| Note: OR= Odds ratio; CI= 95% confidence interval; SBP= Systolic blood pressure; DBP= Diastolic blood pressure;  ^a^We added variables with p < 0.01 into forward and backward stepwise regressions and both arrived at the same parsimonious models.  ^b^High risk of fall = Defined using Morse Fall Scale risk score of 55 or more; Polypharmacy= Use of 4 or more chronic medication. | | | | |

**Table S2 Sensitivity Analysis (Stepwise regression)**

| **Association between any change in anti-hypertensive medication and injurious fall (N = 139)** | | | | |
| --- | --- | --- | --- | --- |
|  | Adjusted OR  (95% CI) | P-value | Stepwise Regression^c^  (95% CI) | P-value |
| Any change in anti-hypertensive medication^a^ |  |  |  |  |
| No | 1.00 |  | 1.00 |  |
| Yes | 3.88 (1.23-12.19) | 0.02 | 3.66 (1.19-11.23) | 0.02 |
| Charlson comorbidity index | 1.28 (1.04-1.58) | 0.02 | 1.30 (1.06-1.59) | 0.01 |
| High risk of fall^b^ | 5.73 (1.60-20.51) | 0.01 | 6.47 (1.86-22.50) | 0.03 |
| Medical history – Hypertension | 0.30 (0.05-1.75) | 0.18 |  |  |
|  |  |  |  |  |
| Note: OR= Odds ratio; CI= 95% confidence interval; SBP= Systolic blood pressure; DBP= Diastolic blood pressure;  ^a^Any change in anti-hypertensive medication= An addition of a new class of anti-hypertensive medication or an increase in the dosage of the existing medication or a switch to a new class of anti-hypertensive medication  ^b^High risk of fall = Defined using Morse Fall Scale risk score of 55 or more.; Polypharmacy= Use of 4 or more chronic medication  ^c^We added variables with p < 0.01 into forward and backward stepwise regressions and both arrived at the same parsimonious models. | | | | |
